# Identification of Bioactive Components from *Ruellia tuberosa* L. on Improving Glucose Uptake in TNF-*α*-Induced Insulin-Resistant Mouse FL83B Hepatocytes

**DOI:** 10.1155/2020/6644253

**Published:** 2020-12-01

**Authors:** Jian-Hua Xu, Yangming Martin Lo, Wen-Chang Chang, Da-Wei Huang, James Swi-Bea Wu, Yu-Yuan Jhang, Wen-Chung Huang, Chih-Yuan Ko, Szu-Chuan Shen

**Affiliations:** ^1^Department of Tumor Surgery, The Second Affiliated Hospital of Fujian Medical University, Quanzhou 362000, China; ^2^Institute for Advanced Study, Shenzhen University, Shenzhen 518060, China; ^3^Department of Food Science, National Chiayi University, Chiayi City 60004, Taiwan; ^4^Department of Biotechnology and Food Technology, Southern Taiwan University of Science and Technology, Tainan City 71005, Taiwan; ^5^Graduate Institute of Food Science and Technology, National Taiwan University, Taipei 10672, Taiwan; ^6^Graduate Program of Nutrition Science, National Taiwan Normal University, Taipei 10610, Taiwan; ^7^Graduate Institute of Health Industry Technology, Chang Gung University of Science and Technology, Taoyuan 33303, Taiwan; ^8^Department of Respiratory and Critical Care Medicine, The Second Affiliated Hospital of Fujian Medical University, Quanzhou 362000, China; ^9^Department of Clinical Nutrition, The Second Affiliated Hospital of Fujian Medical University, Quanzhou 362000, China; ^10^School of Public Health, Fujian Medical University, Fuzhou 350122, Fujian, China; ^11^Respiratory Medicine Center of Fujian Province, Quanzhou 362000, China

## Abstract

*Ruellia tuberosa* L. (RTL) has been used as a folk medicine to cure diabetes in Asia. RTL was previously reported to alleviate hyperglycemia, insulin resistance (IR), abnormal hepatic detoxification, and liver steatosis. However, the potential bioactive compounds of RTL have still not been identified. The aim of this study was to investigate the bioactive compounds in RTL ethyl acetate (EA) fractions by using a glucose uptake assay in TNF-*α*-treated mouse FL83B hepatocytes to discover a mechanism by which to improve IR. The bioactive compounds were identified by the high-performance liquid chromatography (HPLC) assay. Using the Sephadex LH20 gel packing chromatography column, the EAF5 fraction was isolated from RTL and significantly increased glucose uptake in TNF-*α*-treated FL83B cells. Moreover, the MCI gel packing chromatography column separated EAF5 into five subfractions and had no significant cytotoxic effect in FL83B cells when treated at the concentration of 25 *μ*g/ml. Among the subfractions, EAF5-5 markedly enhanced glucose uptake in TNF-*α*-treated FL83B cells. The possible bioactive compounds of the EAF5-5 fraction that were identified by the HPLC assay include syringic acid, *p*-coumaric acid, and cirsimaritin. The bioactive compound with the best effect of increasing glucose uptake was *p*-coumaric acid, but its effect alone was not as good as the combined effect of all three compounds of the EAF5-5 fraction. Thus, we speculate that the antidiabetic effect of RTL may be the result of multiple active ingredients.

## 1. Introduction

Type 2 diabetes mellitus (T2DM) is characterized by insulin resistance (IR), which is caused by insufficient production of insulin or by the ineffectiveness of insulin activity to maintain constant blood sugar. In response to hyperglycemia, the presence of high levels of blood sugar, the body compensatively secretes more blood insulin in increasing concentrations, causing hyperinsulinemia. Once the body presents hyperglycemia and hyperinsulinemia, it can be prone to many complications [1]. Diabetic control and therapy can consist of three parts, including dietary therapy, exercise, and medications. Unfortunately, most T2DM patients will depend on drug treatments, which have adverse side effects. Diabetic drugs have been reported to cause nausea, abdominal pain, or weight gain [2, 3]. Recently, researchers have turned to natural plant ingredients containing phenolic acids or flavonoids. Moreover, various ingredients have been proven to alleviate hyperglycemia or antidiabetic effects [4–6].

*Ruellia tuberosa* L. (RTL) is a herb plant that has been used as folk medicine to cure diabetes in Asian countries for decades. Our previous studies have demonstrated that RTL aqueous or ethanolic extracts can significantly improve glucose uptake in C2C12 myoblasts, alleviate the tumor necrosis factor (TNF)-*α*-induced IR in skeletal muscles, and ameliorate hyperglycemia, IR indices, and aorta dysfunction in high-fat-diet-fed streptozotocin-induced T2DM rats [7, 8]. Additionally, RTL ameliorated abnormal hepatic detoxification, nonalcoholic fatty liver disease, and lipid accumulation in the liver of T2DM rats [9, 10].

Previous studies have identified more than sixty compounds in RTL. The compounds obtained by separation and purification are mainly flavonoids, triterpenes, and steroids also containing long-chain fatty acids, alkaloids, and lignans [11–13]. However, there remains a void in the literature regarding the role of these potent RTL compounds alleviating hepatic glucose uptake. Therefore, we aimed to screen the best glucose uptake activity of TNF-*α*-induced IR in FL83B hepatocytes with ethyl acetate (EA) fractions of RTL by using thin-layer chromatography (TLC) and high-performance liquid chromatography (HPLC) to identify and characterize the specific potent compounds.

## 2. Materials and Methods

### 2.1. Preparation of RTL Extracts

**Figure 1:**
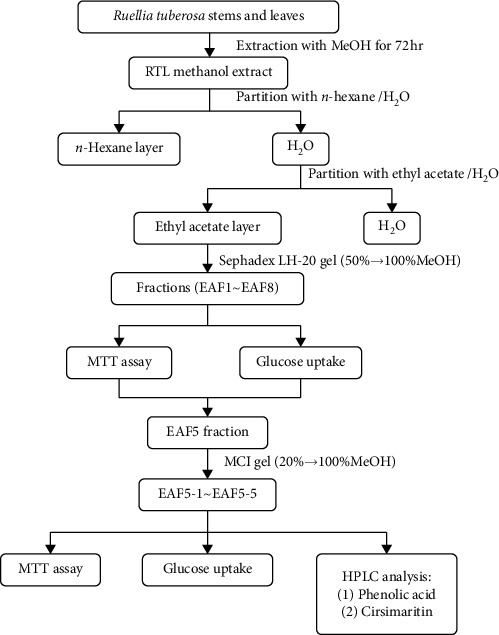
The flowchart for fractionation of *Ruellia tuberosa* Linn. (RTL) extraction.

The extraction protocol was based on our previous methods, with slight modifications [7, 9]. One gram of RTL stems and leaves were extracted with 6 ml distilled water and methanol (1 : 6, *w*/*v*) at 4°C for 72 h and filtered using a cheese cloth. The filtrate was filtered twice through a Whatman No. 1 filter paper and centrifuged at 7,000 × g for 20 min. The supernatant was vacuum concentrated using a rotary evaporator below 40°C. The concentrated methanol extract was dissolved in 400 ml water and subsequently extracted with an equal volume of hexane and EA solvent, and the EA layers were divided through column chromatography using a Sephadex LH-20 column with 200 ml of 50–100% methanol sequentially. The different fractions were then collected from the EA layer (EAF1 to EAF8). The best of glucose uptake hepatocytes of fractions was evaluated and screened, and the EAF5 fractions were divided through column chromatography using an MCI gel column with 200 ml of 20–100% methanol sequentially. The different fractions were then collected from the EAF5 layer (EAF5-1 to EAF5-5), as shown in Figure 1.

### 2.2. TLC

The supernatants were placed onto a silica gel precoated plate (Kieselgel 60 F254, 0.20 mm, Merck, Darmstadt, Germany). The TLC plates were added with a solvent mixture of dichloromethane : methanol : water : acetic acid (10 : 1:0.1 : 0.2, *v*/*v*), followed by immersion into 10% sulfuric acid, and then the mixture was heated. Using the color distribution state of TLC, similar effluents were gathered, and the solvent was drained through a rotary evaporator. Different concentrates were freeze-dried into a powder and kept at −80°C until use.

### 2.3. Cell Culture

The experiments were performed on FL83B mouse hepatocytes (ATCC, Rockville, MD, USA) incubated in F12K medium containing 10% fetal bovine serum (Invitrogen Corporation, Camarillo, CA, USA) in 10 cm Petri dishes at 37°C and 5% CO_2_. The experiments were conducted on cells that were 80–90% confluent.

### 2.4. TNF-*α* Induction of IR in FL83B Cells

IR was induced according to a method described previously [7, 14]. FL83B cells were seeded in 10 cm dishes and incubated at 37°C for 48 h to reach 80% confluences. Serum-free medium containing recombinant mouse TNF-*α* and different RTL fractions (25 *μ*g/ml) was incubated for 16 h at 37°C to induce IR.

### 2.5. Uptake of Fluorescent 2-[N-(7-Nitrobenz-2-oxa-1,3-diazol-4-yl) amino]-2-deoxy-d-glucose (2-NBDG) in FL83B cells

The FL83B cells were seeded in 10 cm dishes and then incubated at 37°C for 48 h to achieve 80% confluences. Serum-free medium containing 20 ng/ml recombinant mouse TNF-*α* was added before incubating for 16 h to induce IR. The cells were then transferred to another F12K medium containing 5 mm glucose, without (basal) or with 200 *μ*m insulin and 10 *μ*l different RTL fractions and incubated for 30 min at 37°C. An assay of glucose uptake was then performed as described previously [7]. The fluorescence intensity of the cell suspension was evaluated using flow cytometry (FACScan, Becton Dickinson, Bellport, NY, USA) at an excitation wavelength of 488 nm and an emission wavelength of 542 nm. Fluorescence intensity reflected the cellular uptake of 2-NBDG.

### 2.6. Chemical Profiles

The total phenolic and flavonoid contents of EA extracts were determined by the HPLC assay [15, 16]. Gallic acid, protocatechuic acid, vanillic acid, syringic acid, caffeic acid, sinapic acid, ferulic acid, *p*-coumaric acid, and cinnamic acid were chosen as standards for phenols, whereas cirsimaritin was chosen as the standard for flavonoids. The samples (10 mg) were dissolved in 1 ml methanol and filtered through a 0.22 *μ*m filter. The HPLC system was equipped with a diode array detector (Shimadzu, Kyoto, Japan) and Varian Polaris C18 column (5 *µ*m, 250 mm × 4.6 mm; Agilent, CA, USA). The injection volume was 10 *μ*l with a flow rate of 1.0 ml/min. For the phenolic acid assay, the mobile phases were methanol (eluent A) and acetic acid (eluent B) in the following gradient elutions: 5% (A), 95% (B) in 0–5 min; 5%–20% (A), 95%–90% (B) in 5–10 min; 20%–40% (A), 90%–60% (B) in 10–20 min; 40%–60% (A), 60%–40% (B) in 20–30 min; and 60%–100% (A), 40%–0% (B) in 30–40 min. The samples were detected by a UV detector (280 nm). For the flavonoid assay, the mobile phases were methanol (eluent A) and acetic acid (eluent B) in the following gradient elutions: 50%–100% (A), 50%–0% (B) in 0–25 min. The samples were detected by using a UV detector (270 nm). The standards for phenolic acids and cirsimaritin (purchased from Sigma, St Louis, MO, USA) had a coefficient of determination (*r*^2^) that was greater than 0.995.

### 2.7. Statistical Analyses

Values are presented as the mean ± standard deviation using SPSS version 22.0 (SPSS Inc., Chicago, IL, USA) by one-way analysis of variance (ANOVA) and Duncan's new multiple range tests. *P* < 0.05 was considered statistically significant.

## 3. Results

### 3.1. Effect of Different EA Fractions from RTL on Glucose Uptake in FL83B Mouse Hepatocytes

The different RTL fractions from the EA layers (EAF1 to EAF8) were collected through TLC analysis (Supplementary Figure S1). When the concentration of each fraction was less than 25 *μ*g/ml, the cell survival rate was greater than 80% and did not inhibit cell growth (Supplementary Figure S2). Thus, the concentration of 25 *μ*g/ml was used for the follow-up glucose uptake evaluation.

**Figure 2:**
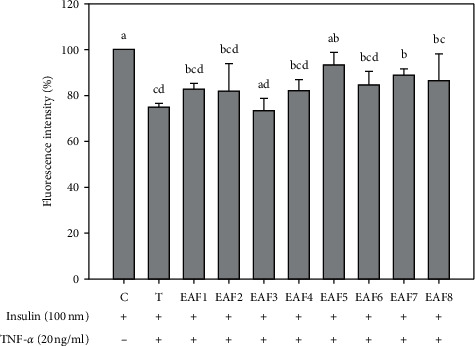
Effects of RTL-EA fractions on glucose uptake in mouse FL83B hepatocytes. Each value is mean ± SD (*n* = 3). C (control): FL83B cells incubated with F-12K medium. T (TNF-*α* treatment): FL83B cells incubated with F-12K medium containing TNF-*α* (20 ng/ml) for 16 hours to induce insulin resistance. EAFs: TNF-*α* induced insulin resistance and then coincubated with RTL-EA fractions (25 *µ*g/ml) for 30 min. ^*∗*^Significantly different from control (*P* < 0.05).

An evaluation of the 2-NBDG uptake was performed to assess the improvement of glucose uptake in FL83B hepatocytes. Compared with the TNF-*α*-treated group, the fluorescence content of the two fractions of EAF5 and EAF7 was significantly increased, and the fluorescence intensity was 93.3 ± 5.4% and 88.9 ± 2.8% (*P* < 0.05), respectively. The EAF5-treated group demonstrated the best improvement in glucose uptake in the IR-induced FL83B hepatocytes. Thus, EAF5 was used for the following examinations (Figure 2).

### 3.2. Effect of Different EAF5 Fractions from RTL on Glucose Uptake in FL83B Mouse Hepatocytes

**Figure 3:**
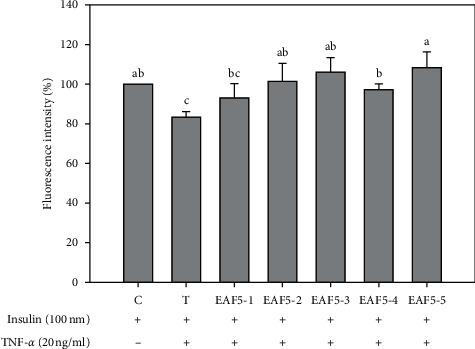
Effect of RTL-EAF5 fractions on glucose uptake in mouse FL83B hepatocytes. Each value is mean ± SD (*n* = 3). C (control): FL83B cells incubated with F-12K medium. T (TNF-*α* treatment): FL83B cells incubated with F-12K medium containing TNF-*α* (20 ng/ml) for 16 hours to induce insulin resistance. EAF5s: TNF-*α* induced insulin resistance and then coincubated with RTL-EAF5 fractions (25 *µ*g/ml) for 30 min. ^*∗*^Significantly different from control (*P* < 0.05).

The different EAF5 fractions (EAF5-1–EAF5-5) were collected through TLC analysis (Supplementary Figure S3). MTT assay showed that the optimal concentration was 25 *μ*g/ml for cell survival of EAF5 subfractions (Supplementary Figure S4). The EAF5-5 fraction significantly increased fluorescence intensity of hepatocytes (108.4 ± 7.7%), compared with the TNF-*α*-induced IR group (*P* < 0.05; Figure 3).

### 3.3. Bioactive Components of RTL

Nine standards of phenolic acid, including gallic acid, protocatechuic acid, vanillic acid, syringic acid, caffeic acid, sinapic acid, ferulic acid, *p*-coumaric acid, and cinnamic acid, and retention time (10.66, 17.15, 23.06, 23.66, 24.09, 27.70, 28.24, 28.78, and 35.27 min, respectively) are shown in Supplementary Figure S5. The standard of cirsimaritin was examined by HPLC, and the retention time was 16.91 min, detail shown in Supplementary Figure S6. EAF5-5 contains 27.3 ± 1.4 *µ*g/g syringic acid, 95.0 ± 2.5 *µ*g/g *p*-coumaric acid, and 805.5 ± 24.8 *µ*g/g cirsimaritin (Table 1).

### 3.4. Evaluation of Bioactive Components from RTL on Glucose Uptake in IR of FL83B Hepatocytes

**Figure 4:**
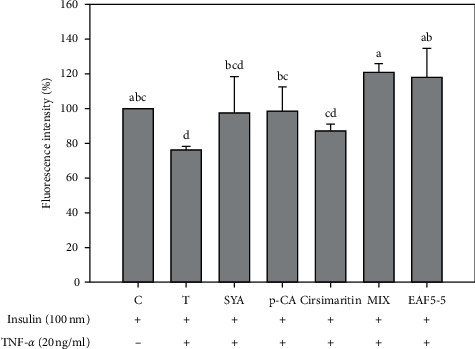
Effects of bioactive components in RTL on glucose uptake in mouse FL83B hepatocytes. Each value is mean ± SD (*n* = 3). C (control): FL83B cells incubated with F-12K medium. T (TNF-*α* treatment): FL83B cells incubated with F-12K medium containing TNF-*α* (20 ng/ml) for 16 hours to induce insulin resistance. SYA: syringic acid; p-CA: *p*-coumaric acid; MIX: syringic acid + *p*-coumaric acid + cirsimaritin. ^*∗*^Significantly different from control (*P* < 0.05).

The fluorescence intensity of syringic acid, *p*-coumaric acid, and cirsimaritin was 97.4 ± 20.8%, 98.8 ± 13.7%, and 87.1 ± 4.3%, respectively, and among them, *p*-coumaric acid has the most significant ability to improve glucose uptake, which can increase by about 22%, compared with the TNF-*α* group (*P* < 0.05; Figure 4).

The fluorescence intensity of the three bioactive components mixed (the mixing ratio was equal to the ratio contained in EAF5-5) and EAF5-5 was 120.9 ± 5.1% and 118.1 ± 16.5%, which were significantly increased by 45% and 42%, respectively, compared to the TNF-*α* group (*P* < 0.05; Figure 4).

## 4. Discussion

In this study, two columns, Sephadex LH-20 gel and MCI gel, were used to separate the bioactive components of the EA layer of RTL. The IR-induced FL83B hepatocytes model was used to screen out the distinguishing substances in the RTL that may improve glucose uptake, and then the active ingredients were identified by HPLC. Our results showed that in the first chromatography column of the EA layer of RTL, the EAF5 fraction significantly increased the glucose uptake of liver cells that induce IR by TNF-*α*; thus, EAF5 was selected for the second chromatography column. EAF5-5 demonstrated the best potential activity for improving cellular glucose uptake based on the activity evaluation of the three types of bioactive phenolic acid and flavonoid compounds—syringic acid, *p*-coumaric acid, and cirsimaritin—which were identified by HPLC analysis. It has been suggested that phenolic compounds can significantly improve the activity of cellular glucose uptake, at least partially. Our results show that among the three active ingredients, *p*-coumaric acid is the most effective for increasing glucose uptake, but its effect is not as good as the combined activity of all three compounds and the EAF5-5 fraction.

An investigation of the cytotoxicity of the EA fractions in the cells was necessary before evaluating the cellular glucose uptake activity. In this study, when the concentration of all EA fractions from RTL was greater than 200 *μ*g/ml, it significantly inhibited cell growth in FL83B mouse hepatocytes. However, when the concentration was less than 25 *µ*g/ml of each fraction, the cell survival rate was more than 80%, which indicates that there was no significant inhibition of cell growth (Supplementary Figures S2 and S4). Hence, a concentration of 25 *μ*g/ml was used for each EAF fraction during the subsequent glucose uptake evaluation. Similarly, it was evident in our previous study that the concentration of RTL improved glucose uptake in mouse C2C12 myoblasts [7].

Administration of RTL fractions of n-hexane and EA in diabetic rabbits lowered the animals' blood sugar levels [17]. Moreover, the best antioxidant activity result was reported from an evaluation of RTL extraction using methanol and EA [18]. RTL fractions of EA can effectively improve the blood sugar level in diabetes through their antioxidant capacity, as EA fractions speculatively contain high amounts of polyphenols and triterpenoids [17]. Another study indicated that the active compound of the EA fraction may be a polyphenol called cirsimaritin [19]. Our findings seem to be consistent with the above studies.

In this study, two phenolic acids, syringic acid and *p*-coumaric acid, were identified by HPLC, which had not been identified from RTL in the past. Previously, vanillic acid was isolated from an RTL extraction utilizing EA [20]. However, our present study did not identify vanillic acid from the fractions of RTL. It is speculated that when this compound cannot be identified, it is due to too little content or the extraction method that was used.

However, previous studies have confirmed that syringic acid and *p*-coumaric acid have many physiological activities, such as antioxidation, anticancer, and antibacterial [21–25]. Moreover, the effects of both of these compounds in treating diabetes have been demonstrated [26]. The administration of syringic acid in alloxan-induced diabetic rats for 30 days showed increased insulin, glycogen, glucose 6-phosphate dehydrogenase, and C-peptide contents, as well as decreased blood sugar, HbA1c, and glucokinase, which are associated with an improvement in diabetic symptoms. Additionally, in histopathological sections, syringic acid was found to reduce pancreatic damage caused by alloxan and stimulate *β*-cell regeneration, thus demonstrating its potential to treat diabetes [27, 28].

Yoon et al. [29] demonstrated that *p*-coumaric acid can improve IR and the lipid metabolism of skeletal muscle cells. The administration of *p*-coumaric (100 mg/kg BW) in streptozotocin-induced diabetic rats for 30 days can reduce the fasting blood glucose level and HbA1c, as well as increase the insulin level. In addition, *p*-coumaric has been found to have excellent antioxidant ability and can enhance the activity of antioxidant enzymes (catalase, superoxide dismutase, and glutathione peroxidase) of the kidney and liver [23].

Based on the above results, it is speculated that the two phenolic acids––syringic acid and *p*-coumaric acid—may be the active ingredients for the EAF5-5 fraction to improve glucose uptake in mouse FL83B liver cells and will therefore be developed into drugs or dietary supplements for the treatment of diabetes in the future.

On the other hand, flavonoids isolated from natural substances have been proven to have a variety of physiological effects, such as antioxidation, lowering blood lipids, or antidiabetics [30–32]. RTL contains a variety of flavonoids, such as cirsimaritin, cirsimarin, cirsiliol 4′-glucoside, sorbifolin, or pedalitin [13, 20]. At present, there are no animal experiments to explore the hypoglycemic effect of cirsimaritin, but cirsimaritin has been found in many natural products with antidiabetic properties [33, 34]. It is speculated that cirsimaritin may be one of the main active ingredients of flavonoids from the EAF5-5 fraction to improve the glucose uptake capacity of insulin-resistant FL83B hepatocytes.

In this study, three components (syringic acid, *p*-coumaric acid, and cirsimaritin) that were isolated from the EAF5-5 fraction were shown to improve cellular glucose uptake, with *p*-coumaric acid demonstrating the best effect. However, the increase in cellular glucose update is highest when all three compounds were used. We speculated that the hypoglycemic effect of RTL may be the result of multiple active ingredients. One theory has pointed out that if plants can be used to treat diabetes, then their effects can be multiplied by using them in higher concentrations and in combination with the chemical components that are contained in a variety of medicinal plants to cure diabetes through distinct mechanisms [35]. Thus, the mechanisms by which RTL improves glucose uptake needs further study.

## 5. Conclusions

Sephadex LH20 gel and MCI gel were used to separate the EA layer of RTL. An evaluation of the glucose uptake in IR-induced FL83B hepatocytes showed that EAF5-5 demonstrated the best activity for improving cell glucose uptake. These activities were eventually linked by HPLC to two phenolic compounds—syringic acid and *p-*coumaric acid—and the flavonoid compound cirsimaritin. Among the three compounds that significantly improved the activity of cellular glucose uptake, *p*-coumaric acid was the active ingredient with the best effect of increasing glucose uptake although its effect was still not as good as the combined effect of all three compounds and the EAF5-5 fraction. Thus, we speculate that the antidiabetic effect of RTL may be the result of multiple active ingredients, but additional research is needed to confirm this.

## Figures and Tables

**Table 1:** Retention time and contents of phenolic acid and flavanoid from the RTL-EAF5 fraction.

Standard	Retention time (min)	EAF5-1 (*µ*g/g)	EAF5-2 (*µ*g/g)	EAF5-3 (*µ*g/g)	EAF5-4 (*µ*g/g)	EAF5-5 (*µ*g/g)
Phenolic acid						
Syringic acid	23.66	—	—	—	6.7 ± 0.9	27.3 ± 1.4
*p*-Coumaric acid	28.78	—	—	—	—	95.0 ± 2.5
Flavanoid						
Cirsimaritin	16.91	—	—	—	—	805.5 ± 24.8

—, not detected. Each value is mean ± SD (*n* = 3).

## Data Availability

All the data used to support the findings of this study are included within the article.

## Supplementary Materials

Supplementary Materials Figure S1: thin layer chromatography of eight fractions from RTL-EA by Sephadex LH20 gel column chromatography. Figure S2: concentration effect of RTL-EA fractions on the cell viability of mouse FL83B hepatocytes. Figure S3: thin layer chromatography of five fractions from RTL-EAF5 by MCI gel column chromatography. Figure S4: concentration effect of RTL-EAF5 fractions on the cell viability of mouse FL83B hepatocytes. Figure S5: HPLC chromatogram of phenolic acids from RTL-EAF5-5 fraction. Figure S6: HPLC chromatogram of RTL-EAF5-5 fraction.
