# Supplementary material for: Identification of Bioactive Components from Ruellia tuberosa L. on Improving Glucose Uptake in TNF-α-Induced Insulin-Resistant Mouse FL83B Hepatocytes
Source: Evid Based Complement Alternat Med. 2020 Dec 1;2020:6644253. doi: 10.1155/2020/6644253 (PMC7723485; doi:10.1155/2020/6644253)
Supplement: Supplementary Materials — Figure S1: thin layer chromatography of eight fractions from RTL-EA by Sephadex LH20 gel column chromatography. Figure S2: concentration effect of RTL-EA fractions on the cell viability of mouse FL83B hepatocytes. Figure S3: thin layer chromatography of five fractions from RTL-EAF5 by MCI gel column chromatography. Figure S4: concentration effect of RTL-EAF5 fractions on the cell viability of mouse FL83B hepatocytes. Figure S5: HPLC chromatogram of phenolic acids from RTL-EAF5-5 fraction. Figure S6: HPLC chromatogram of RTL-EAF5-5 fraction. [file 6644253.f1.pdf]

Supplementary data

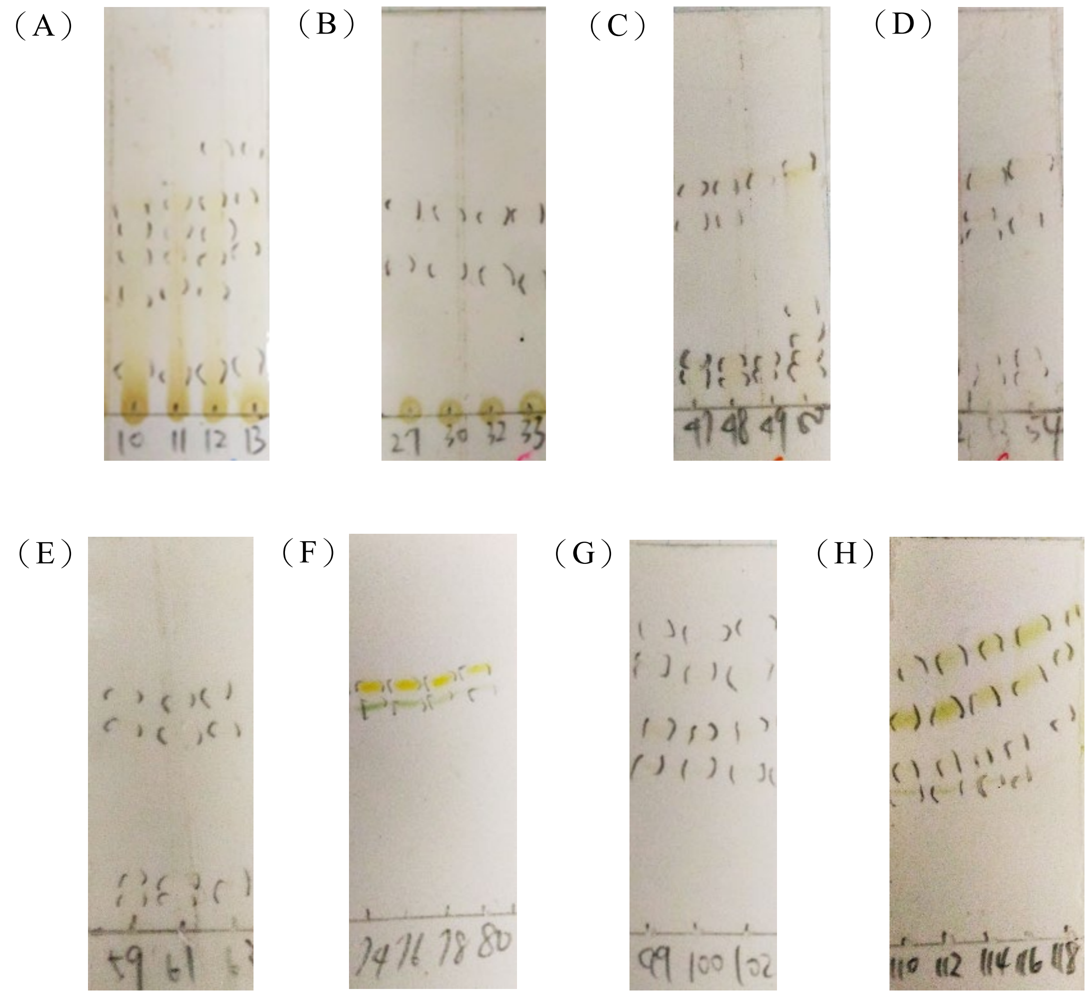

Figure S1. Thin layer chromatography of eight fractions from RTL-EA by Sephadex LH-20 gel column chromatography. (A) RTL-EA fraction 1 (EAF1), (B) RTL-EA fraction 2 (EAF2), (C) RTL-EA fraction 3 (EAF3), (D) RTL-EA fraction 4 (EAF4), (E) RTL-EA fraction 5 (EAF5), (F) RTL-EA fraction 6 (EAF6), (G) RTL-EA fraction 7 (EAF7), (H) RTL-EA fraction 8 (EAF8).

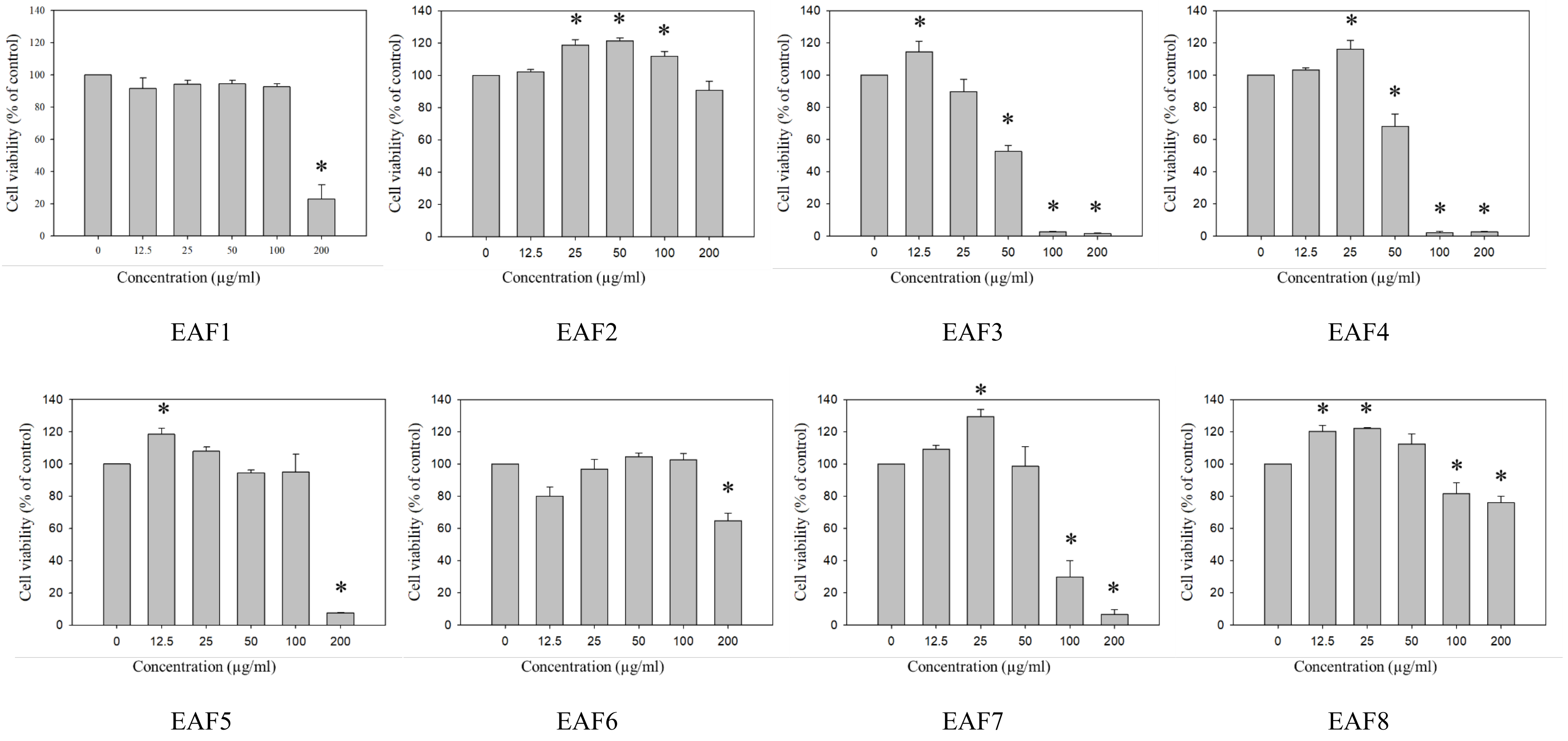

Figure S2. Concentration effect of RTL-EA fractions on the cell viability of mouse FL83B hepatocytes.

Cell Viability (%)= (Sample group − Blank group) / (Control group − Blank group)×100%

*significantly different from control (0 μg/ml). ($P < 0.05$).

Each value is means±SD (n=3).

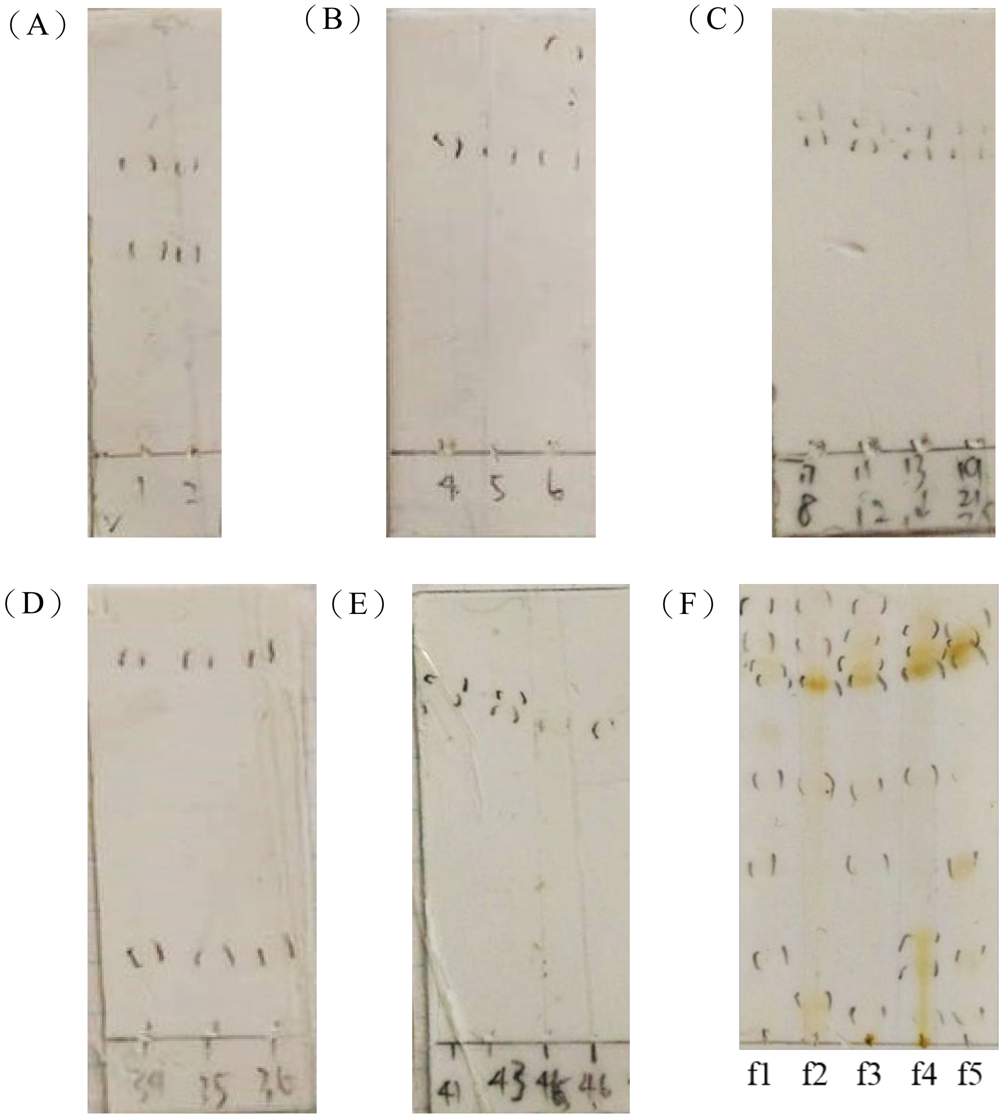

Figure S3. Thin layer chromatography of five fractions from RTL-EAF5 by MCI gel column chromatography.

(A) RTL-EA fraction 5-1 (EAF5-1) detected under UV; (B) RTL-EA fraction 5-2 (EAF5-2) detected under UV; (C) RTL-EA fraction 5-3 (EAF5-3) detected under UV; (D) RTL-EA fraction 5-4 (EAF5-4) detected under UV; (E) RTL-EA fraction 5-5 (E AF5-5) detected under UV; (F) EAF5-1- EAF5-5 detected by chemical reagent, H_2SO_4.

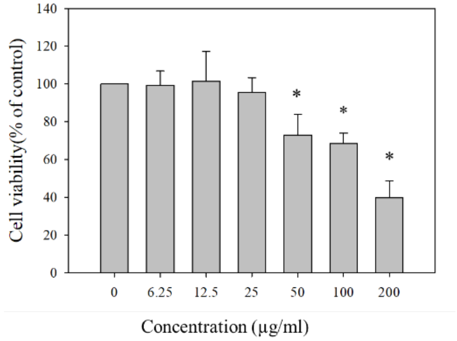

EAF5-1

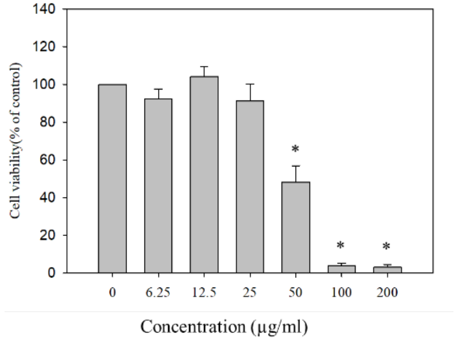

EAF5-2

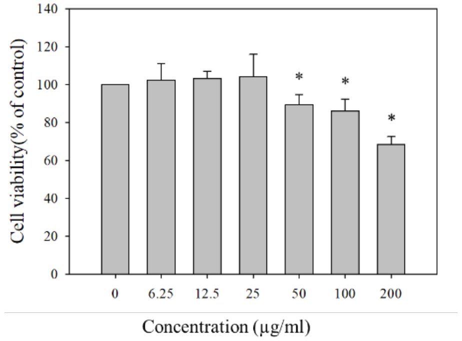

EAF5-3

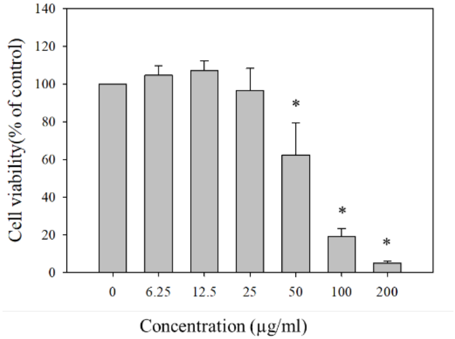

EAF5-4

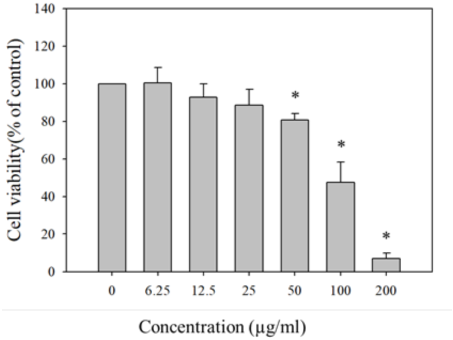

EAF5-5

Figure S4. Concentration effect of RTL-EAF5 fractions on the cell viability of mouse FL83B hepatocytes.

Cell Viability (%)= (Sample group–Blank group) / (Control group–Blank group)×100%

*significantly different from control (0 μg/ml). ($P < 0.05$).

Each value is means±SD (n=3).

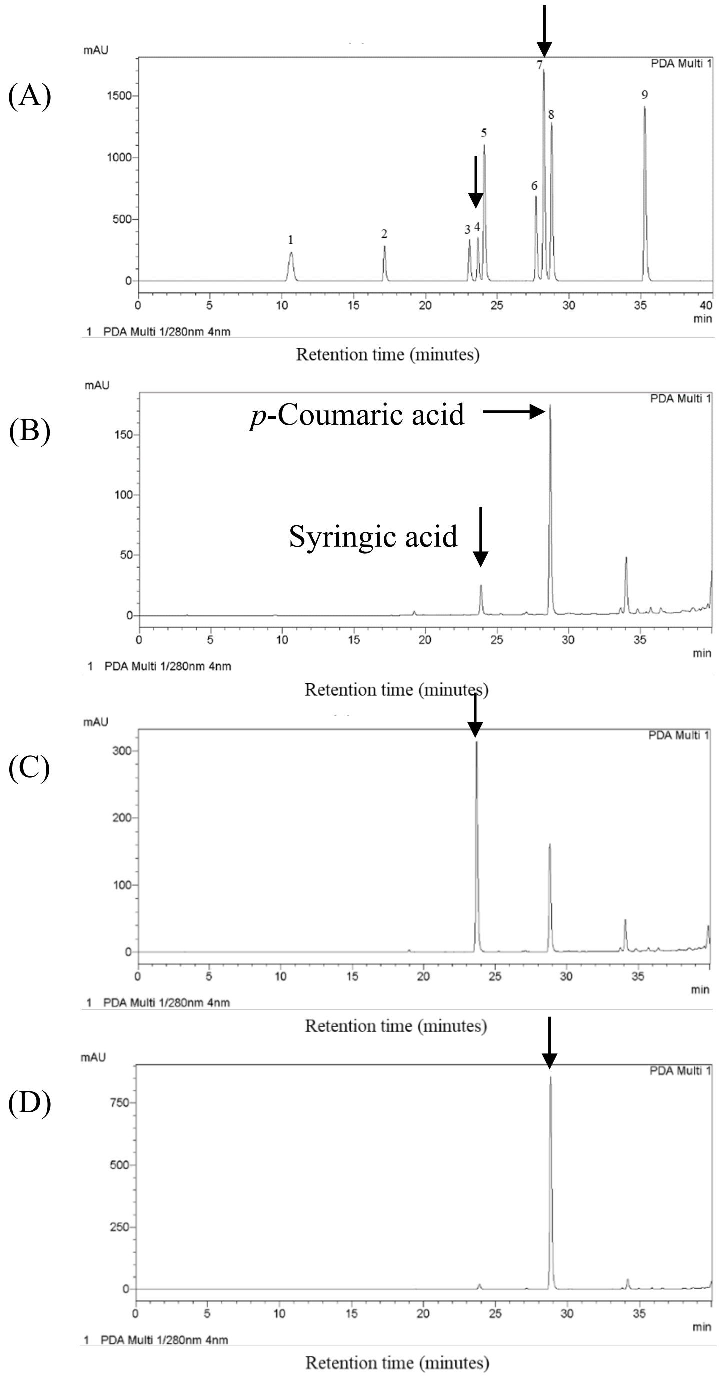

Figure S5. HPLC chromatogram of phenolic acids from RTL-EAF5-5 fraction.
(A) Phenolic acids [Peak 1: gallic acid (GA), Peak 2: protocatechuic acid (PA), Peak 3: vanillic acid (VA), Peak 4: syringic acid (SYA), Peak 5: caffeic acid (CFA), Peak 6: sinapic acid (SIA), Peak 7: ferulic acid (FA), Peak 8: p-coumaric acid (p-CA), Peak 9: cinnamic acid (CA)], (B) RTL-EAF5-5 9 mg/ml, (C) RTL-EAF5-5 9 mg/ml + syringic acid (SYA) 200 µg/ml, (D) RTL-EAF5-5 9 mg/ml + p-Coumaric acid (PCA) 200 µg/ml.

HPLC conditions:

Column: Varian Polaris C18 (5 μm, 250 mm x 4.6 mm).

Mobile phase: 100% methanol (A) / 0.1% aqueous acetic acid solution (B).

Gradient Elution: 0-5 min: 5% (A), 95% (B); 5-10 min: 5%-20% (A), 95%-90% (B); 10-20 min: 20% - 40% (A), 90%–60% (B); 20-30 min: 40%-60% (A), 60%-40% (B); 30- 40 min: 60%-100% (A),40% – 0% (B).

Inject volume: 10 μl.

Flow rate: 1 ml/min.

Absorbance: 280 nm.

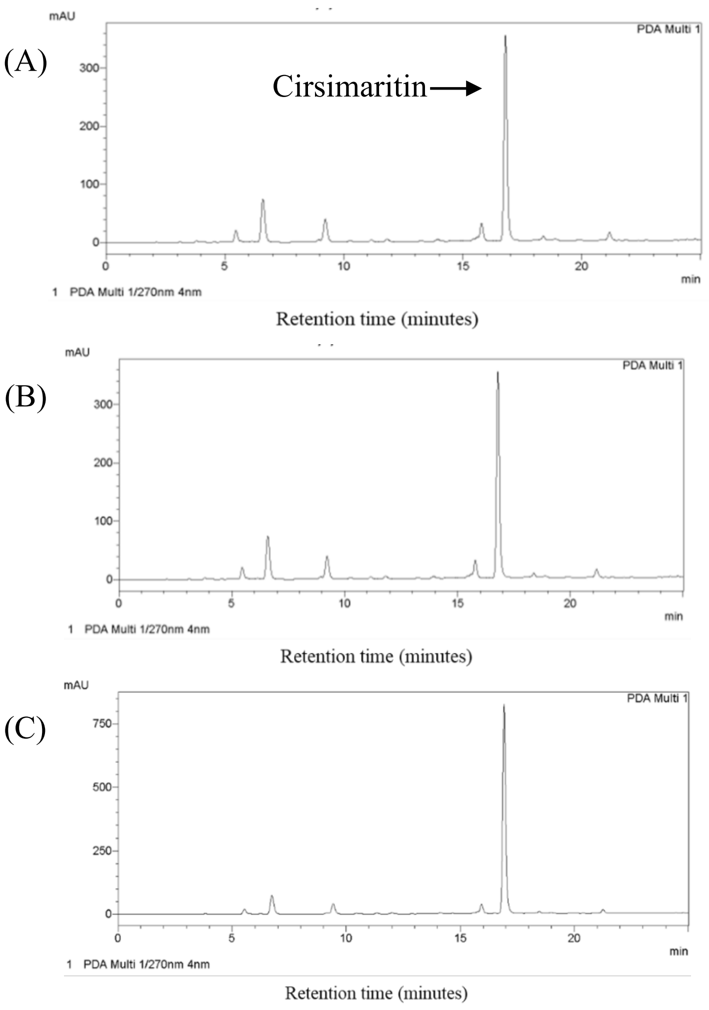

Figure S6. HPLC chromatogram of RTL-EAF5-5 fraction.
(A) Cirsimaritin, (B) RTL-EAF5-5 9000 mg/ml, (C) RTL-EAF5-5 9000 mg/ml + Cirsimaritin 200 μg/ml.

HPLC conditions:
Column: Varian Polaris C18 (5μm, 250mm x 4.6mm).
Mobile phase: 100% methanol (A)/0.1% aqueous acetic acid solution (B).
Gradient Elution: 0-25 min: 50%-100% (A), 50%- 0% (B).
Inject volume: 10μl.
Flow rate: 1ml/min.
Absorbance: 270 nm.
